# Nuclear Translocation of SGPP-1 and Decrease of SGPL-1 Activity Contribute to Sphingolipid Rheostat Regulation of Inflammatory Dendritic Cells

**DOI:** 10.1155/2017/5187368

**Published:** 2017-12-11

**Authors:** Anja Schwiebs, Dominique Thomas, Burkhard Kleuser, Josef M. Pfeilschifter, Heinfried H. Radeke

**Affiliations:** ^1^Institute of General Pharmacology and Toxicology, pharmazentrum frankfurt/ZAFES, Hospital of the Goethe University, Frankfurt, Germany; ^2^Institute of Clinical Pharmacology, pharmazentrum frankfurt, Hospital of the Goethe University, Frankfurt, Germany; ^3^Institute of Nutritional Science, University of Potsdam, Potsdam, Germany

## Abstract

A balanced sphingolipid rheostat is indispensable for dendritic cell function and survival and thus initiation of an immune response. Sphingolipid levels are dynamically maintained by the action of sphingolipid enzymes of which sphingosine kinases, S1P phosphatases (SGPP-1/2) and S1P lyase (SGPL-1), are pivotal in the balance of S1P and sphingosine levels. In this study, we present that SGPP-1 and SGPL-1 are regulated in inflammatory dendritic cells and contribute to S1P fate. TLR-dependent activation caused SGPL-1 protein downregulation with subsequent decrease of enzymatic activity by two-thirds. In parallel, confocal fluorescence microscopy revealed that endogenous SGPP-1 was expressed in nuclei of naive dendritic cells and was translocated into the cytoplasmatic compartment upon inflammatory stimulation resulting in dephosphorylation of S1P. Mass spectrometric determination showed that a part of the resulting sphingosine was released from the cell, increasing extracellular levels. Another route of diminishing intracellular S1P was possibly taken by its export via ATP-binding cassette transporter C1 which was upregulated in array analysis, while the S1P transporter, spinster homolog 2, was not relevant in dendritic cells. These investigations newly describe the sequential expression and localization of the endogenous S1P regulators SGPP-1 and SGPL-1 and highlight their contribution to the sphingolipid rheostat in inflammation.

## 1. Introduction

Besides its migratory effects, S1P importantly contributes to inflammatory processes by intracellular signaling [1]. Its production, metabolism, and transportation define the sphingolipid rheostat which plays a critical role in dendritic cell (DC) survival as well as cytokine secretion and antigen capture [2–4]. S1P can function intracellularly as a second messenger or be secreted out of the cell and act extracellularly by signaling through S1P receptors in autocrine and paracrine manners. We and others demonstrated that high extracellular concentrations of S1P lead to the differentiation of DC populations producing less IL-12p70 after TLR4 stimulation [3, 5] and a limited capacity to initiate Th1 responses [6]. Deletion of one major S1P-producing enzyme sphingosine kinase 1 (Sphk1) in dendritic cells leads to a reduction of cell survival and metabolic activity [2].

It has been postulated that S1P levels inside cells are tightly regulated by the balance between the S1P synthesizing enzymes Sphk1 and Sphk2 and by metabolizing/degrading enzymes like phosphatases and S1P lyase (SGPL-1). SGPL-1 is thereby irreversibly degrading S1P into hexadecenal and phosphoethanolamine [7]. S1P phosphatases 1 and 2 (SGPP-1/2) reversibly metabolize S1P into sphingosine [8, 9].

Initially described by Spiegel's group, the S1P phosphatase 1 is highly specific toward long-chain sphingoid base phosphates and degrades S1P, dihydro-S1P, and phyto-S1P [9–11]. The transfection of a mammalian SGPP-1 into NIH 3T3 fibroblasts decreased S1P levels, increased ceramide levels, and diminished cell survival [10]. Human SGPP-1 is expressed in most tissues, with the strongest levels found in highly vascularized tissues [12]. SGPP-1 is downregulated in human gastric cancer tissues and plays a role in invasion and migration in gastric cancer cells [13]. SGPP-1 contributes to ER stress-induced autophagy in human breast adenocarcinoma MCF7 cells [14]. Its biological function has also a prominent role in keratinocyte development by contributing to an imbalance between S1P and ceramides [15].

Despite the obvious importance of S1P-degrading pathways, only a sparse set of investigations focuses on S1P phosphatases during cellular differentiation and cancer, with actually no study dealing with SGPP-1 in immune cells. Previously, we have described a dominant loss of S1P upon TLR activation on dendritic cells [2]. Thus, in this consecutive study, we investigated S1P dephosphorylating enzyme SGPP-1 and the terminally degrading enzyme SGPL-1, as well as other possible mechanisms to reduce intracellular S1P levels in inflammatory dendritic cells.

## 2. Methods

### 2.1. Isolation, Differentiation, and Stimulation of Bone Marrow Cells

Isolation, differentiation, and stimulation of bone marrow cells were performed as described before [2]. In brief, female C57BL/6 wild-type mice (Janvier, Saint-Berthevin Cedex, France) were used. All animals were bred at the local animal facility under specific pathogen-free conditions. All animal experiments were performed in accordance with the German animal welfare law and had been declared to the Animal Welfare Officer as the chairperson of the ethical oversight committee of the Goethe University Frankfurt/Main. The animal housing facility was licensed by the local authorities of the Regierungspraesidium Darmstadt (Az: 32.62.1). The methods used to euthanize the animals humanely were consistent with the recommendations of the AVMA Guidelines for the Euthanasia of Animals. Euthanized animal bone marrow was isolated from the tibia and femur and washed, and erythrocytes were lysed. Cells were differentiated in RPMI 1640 GlutaMax medium (Thermo Fisher Scientific, Massachusetts, USA) supplemented with 10% FCS, 100 IU/ml penicillin, 100 *μ*g/ml streptomycin, 10 mM HEPES (Sigma-Aldrich, Steinheim, Germany), 1 mM sodium pyruvate, and 50 *μ*M 2-*β*-ME (Thermo Fisher Scientific, Massachusetts, USA). GM-CSF-differentiated cells have been supplemented with 40 ng/ml GM-CSF for seven days (CD11c^+^ DCs). Flt3 differentiation has been performed by the addition of 200 ng/ml Flt3 ligand and 20 ng/ml GM-CSF for ten days (CD103^+^ cDCs). Differentiated bone marrow-derived DCs were then harvested, seeded without FCS, and stimulated with 1 *μ*g/ml LPS from *Escherichia coli* O127:B8 (Sigma-Aldrich, Steinheim, Germany) or left untreated for the indicated time points. After stimulation, cells or cell pellet was used for RNA isolation, protein extraction, lipid extraction, or stainings.

### 2.2. Isolation and Differentiation of Human PBMCs

Isolation of PMCs from buffy coats was performed according to Nair et al. [16].

In brief, density centrifugation was performed using Ficoll (GE Healthcare, Uppsala, Sweden). Isolated PBMCs have been plated at a density of 2 × 10^8^ cells per dish, and supernatant has been discarded upon 2 h of plastic adherence. Subsequently, cells were differentiated in RPMI 1640 GlutaMax medium (Thermo Fisher Scientific, Massachusetts, USA) supplemented with 10% FCS, 100 IU/ml penicillin, 100 *μ*g/ml streptomycin, 10 mM HEPES (Sigma-Aldrich, Steinheim, Germany), 1 mM sodium pyruvate, and 50 *μ*M 2-*β*-ME (Thermo Fisher Scientific, Massachusetts, USA) supplemented with 40 ng/ml recombinant human GM-CSF (PeproTech, NJ, USA) and human IL-4 (PeproTech, NJ, USA) with an additional medium exchange after 4 days. Differentiated cells were harvested by cell scraping and transferred to tissue-treated 8-well chambered cover slides (Ibidi, Martinsried, Germany) for fluorescence microscopy staining.

### 2.3. Western Blotting

For Western blot analysis, pelleted cells were lysed in a buffer containing 10 mM HEPES-KOH, 10 mM KCl, 0.1 mM EDTA, 0.1 mM EGTA, 0.5 mM NaF, 1 mM Na3VO4, and 1x complete™ protease inhibitor cocktail (Roche Diagnostics, Mannheim, Germany) for 10 min. The cytosolic fraction was used after pelleting the nuclear fraction by centrifugation at 13,000 ×g for 10 min at 4°C. The nuclear fraction was additionally lysed with a buffer containing 20 mM HEPES-KOH, 400 mM NaCl, 1 mM EDTA, 1 mM EGTA, 0.5 mM NaF, 1 mM Na3VO4, and a 1x complete protease inhibitor for 10 min and centrifuged for 10 min at 10,000 ×g at 4°C. Protein concentration was determined by BCA (Thermo Fisher Scientific, Massachusetts, USA), according to the manufacturer's instructions. Whole cell extracts and cell fractions were used for detection with anti-SGPL-1 (ab56183), anti-SGPP-1 (ab108435), anti-MRP-1 (ab32574) (Abcam, Cambridge, UK), and anti-*β*-actin (A5441) (Sigma-Aldrich, Steinheim, Germany) after SDS-PAGE. According to the first antibodies, the second antibody anti-rabbit IgG (GE Healthcare, Little Chalfont, UK) has been used. The protein bands were detected by ECL (Thermo Fisher Scientific, Massachusetts, USA) following the manufacturer's protocol. Quantitative evaluation was performed by densitometry using Quantity one (Bio-Rad, Hercules, CA).

### 2.4. Lipid Extraction and Sphingolipid Analysis by LC-MS/MS

LC-MS/MS was performed as described before [17]. In detail, for the quantification of sphingolipids, cell pellets or supernatants were spiked with an internal standard solution (500 ng/ml; Sph-d7, S1P-d7, Saph-d7, C16:0-Cer-d31, C17-Cer, C18:0-Cer-d3, C18:0-dhCer-d3, C17:0-LacCer, C16:0-LacCer-d3, and C18:0-GluCer-d5; Avanti Polar Lipids, Alabaster, USA; and C24:0-Cer-d4, synthesized by ChiroBlock GmbH, Wolfen, Germany). Afterwards, 150 *μ*l water was added and the analytes were extracted twice with 600 *μ*l methanol : chloroform : HCl (15 : 83 : 2, *v*/*v*/*v*). The collected organic phases were evaporated at 45°C under a gentle stream of nitrogen and reconstituted in 50 *μ*l methanol. For the chromatographic separation, a Luna C18 column (150 mm × 2.0 mm, 5 *μ*m particle size, 100 Å pore size; Phenomenex, Aschaffenburg, Germany) was used. The HPLC mobile phases consisted of water-formic acid (100 : 0.1, *v*/*v*) (A) and acetonitrile-tetrahydrofuran-formic acid (50 : 50 : 0.1, *v*/*v*/*v*) (B). For separation, a gradient program was used at a flow rate of 0.3 ml/min. The initial buffer composition 60% (A)/40% (B) was held for 0.6 min and then within 3.9 min linearly changed to 0% (A)/100% (B) and held for 6.5 min. Subsequently, the composition was linearly changed within 0.5 min to 60% (A)/40% (B) and then held for another 4.5 min. The running time for every sample (injection volume: 15 *μ*l for Cer and dh-Cer determination and 10 *μ*l for the other sphingolipids) was 16 min. MS/MS analyses were performed on an API4000 triple quadrupole mass spectrometer equipped with an APCI (atmospheric pressure chemical ionization) ion source (SCIEX, Darmstadt, Germany) for Cer and dh-Cer determination and with an ESI (electrospray ionization) ion source for the determination of the other sphingolipids. The analysis was done in multiple reaction monitoring (MRM) mode. Two *m*/*z* transitions with a dwell time of 20 ms were recorded for each analyte, the first one for quantification and the second one for qualification, to exclude false positive results. For analysis and quantification, the Analyst Software 1.6 (SCIEX, Darmstadt, Germany) was used and the peak area of each analyte was corrected by the peak area of the corresponding internal standard. Calibration curves were constructed using linear regression with 1/*x* weighting. The coefficient of correlation was at least 0.99. Variations in accuracy were less than 15% over the whole range of calibration, except for the lowest limit of quantification, where a variation in the accuracy of 20% was accepted.

### 2.5. Quantitative Real-Time PCR

Total RNA of pelleted cells was extracted using the peqGOLD Total RNA Kit (peqlab, Erlangen, Germany) as recommended by the manufacturer. RNA concentration was measured using the Nano-Drop 1000 (Thermo Fisher Scientific, Massachusetts, USA) analyzer and was adjusted to 1 *μ*g/*μ*l for first-strand cDNA synthesis using the high-capacity cDNA reverse transcription kit (Life Technologies, Carlsbad, CA). TaqMan® gene expression assays (Life Technologies, Carlsbad, CA) were applied for sgpl-1, sgpp-1, abcc1, abca1, abcg1, and spns2 (Applied Biosystems, Darmstadt, Germany) and for the housekeeping genes csnk2a2 and fbxo38 (Primer Design, Southampton, UK). The Precision FAST Mastermix (Primer Design, Southampton, UK) was used, and quantitative real-time PCR was run at 95°C for 2 min and 40 times at 95°C for 5 s and 60°C for 20 s (7500 Fast Real-Time PCR System, Applied Biosystems, Darmstadt, Germany). The comparative C_T_ method was used for analyzing the results using the mean of the two housekeeping genes as a reference.

### 2.6. Fluorescence Microscopy

Cells were grown on tissue-treated 8-well chambered cover slides (Ibidi, Martinsried, Germany), washed twice with 300 *μ*l ice-cold PBS, and fixed for 4 min with ice-cold methanol on ice. After washing three times with 300 *μ*l PBS, the cells were blocked with 2% BSA-PBS for 1 h and subsequently stained with the first antibody murine or human anti-SGPP-1 (ab108435 and ab129253 from Abcam, Cambridge, UK) or murine anti-SGPL-1 (ab56183 from Abcam) overnight at 4°C. For experiments confirming antibody specificity, murine anti-SGPP-1 antibody was preincubated with the corresponding blocking peptide (ab223885 from Abcam) in a ratio of 1 : 2 for 30 min before addition to the cells. After three washing steps, the second antibody (anti-rabbit, GE Healthcare, UK) and DAPI (4′,6-Diamidine-2′-phenylindole dihydrochloride) solution (Roche Diagnostics, Mannheim, Germany) were applied for 1 hour. Finally, cells were washed and kept in the dark at 4°C until microscopic analysis. For ER tracker colocalization, cells have been fixed with 4% paraformaldehyde instead of methanol and ER tracker Blue-White (Thermo Fisher Scientific, Massachusetts, USA) has been applied before fixation. Confocal laser scanning microscopy was performed with a Zeiss LSM510 Meta system equipped with an inverted Observer Z1 microscope and a Plan-Apochromat 63 × /1.4 oil immersion objective (Carl Zeiss MicroImaging GmbH, Göttingen, Germany).

### 2.7. ABC Transporter Array

A TaqMan array for ABC transporters (Thermo Fisher Scientific, Massachusetts, USA) was performed according to the manufacturer's recommendations. In brief, RNA was isolated and transcribed into cDNA. cDNA was dispensed with a master mix into the 96-well plate array, and thermal cycling conditions have been applied as the following: at 95°C for 20 sec and 40 times at 95°C for 3 s and 60°C for 30 s (7500 Fast Real-Time PCR System, Applied Biosystems, Darmstadt, Germany). The comparative C_T_ method was used for analyzing the results.

### 2.8. SGPL-1 Activity Measurement

SGPL-1 activity measurement was performed by the quantification of (2E)-hexadecenal following derivatization with 2-diphenylacetyl-1,3-indandione-1-hydrazone (DAIH) as described before [18]. In brief, cells were extracted by a cold methanol-chloroform 0.9% NaCl mixture on ice. The organic phase was dried and solved in acetonitrile. Derivatization was performed with a mixture containing 0.6 mg/ml DAIH in acetonitrile and 7% of 2 M HCl at 4°C. The analysis of the aldehyde was conducted with an Agilent 1200 liquid chromatography system coupled to an Agilent 6530 quadrupole/time-of-flight mass spectrometer (both from Waldbronn, Germany). Chromatographic separation was performed on a ZORBAX Eclipse XDB-C18 column.

### 2.9. Statistics

The software GraphPad Prism 6.0 (La Jolla, CA) was used to enter data, display graphs, and perform statistics by Student's *t*-test or others if indicated in the figure legends. Data are represented as means ± SD, and significant values are symbolized as asterisks (^∗^/^∗∗^/^∗∗∗^) which represent *p* values of ≤0.05/≤0.01/≤0.001.

## 3. Results

### 3.1. Regulation of SGPL-1 and SGPP-1 Expression in Inflammatory Dendritic Cells

**Figure 1:**
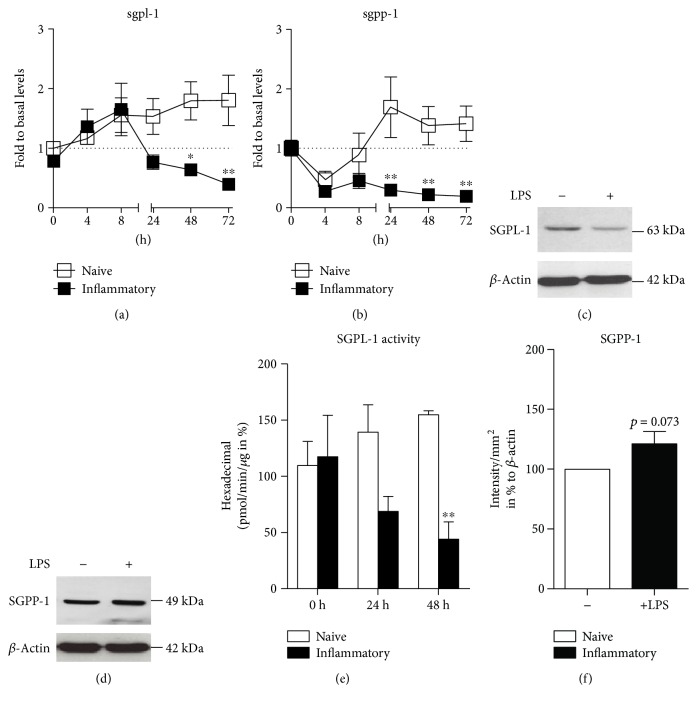
(a, b) Quantitative real-time PCR for *sgpl-1* and *sgpp-1* mRNA in naive and LPS-stimulated dendritic cells over time (*n* = 4). (c) Western blot with anti-SGPL-1 antibody and anti-*β*-actin antibody in naive and LPS-stimulated DCs after 48 h. (d) Quantification of hexadecenal for SGPL-1 activity in naive and LPS-stimulated dendritic cells with QTOF-MS (*n* = 3). (e) Western blot for anti-SGPP-1 antibody and anti-*β*-actin antibody in naive and LPS-stimulated DCs after 48 h (*n* = 3). (f) Quantification of Western blot bands of (e) (statistical test: one sample *t*-test). Data represent mean ± SD; ^∗^*p* ≤ 0.05 and ^∗∗^*p* ≤ 0.01.

To elucidate the sphingolipid rheostat regulation in dendritic cells, we examined how sphingolipid enzyme expression is modulated in DCs. Previously, we have shown that with time, S1P levels are fading in inflammatory cells [2]. Simultaneously, *sphk1* and *sphk2* mRNA levels are not downregulated upon LPS stimulation possibly indicating that modulation of S1P-producing enzymes is likely not contributing to S1P loss in inflammatory DCs [2]. Thus, we examined whether S1P-degrading enzymes SGPL-1 and SGPP-1 play a significant role in S1P fate. Therefore, we isolated bone marrow from wild-type mice and produced GM-CSF-differentiated CD11c^+^ DCs. We observed that *sgpl-1* and *sgpp-1* levels decreased or stayed constantly lower in inflammatory DCs compared to naive DCs over time (Figures 1(a) and 1(b)). Similarly, SGPL-1 protein levels were reduced upon 48 h of inflammatory stimuli compared to naive DCs (Figure 1). The reduced protein levels were mirrored in reduced SGPL-1 enzyme activity which sequentially declined upon inflammatory stimulation by two-thirds indicating that a major force of S1P degradation in inflammatory DCs was dampened (Figure 1). Notably, the reduction of *sgpp-1* mRNA levels did not result in a decrease of SGPP-1 protein levels which rather tended to increase (Figures 1(e) and 1(f)).

### 3.2. SGPP-1 but Not SGPL-1 Is Translocated in Inflammatory Dendritic Cells

**Figure 2:**
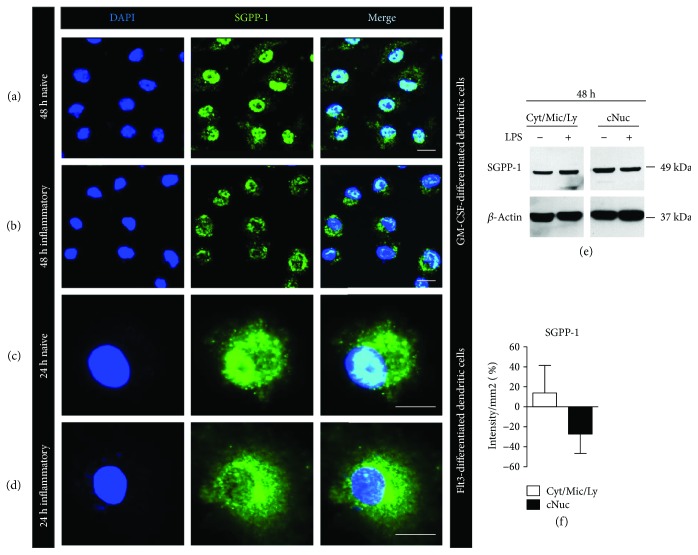
(a, b) Representative pictures of confocal laser microscopy of untreated mature GM-CSF-differentiated CD11c^+^ dendritic cells after 48 h of starvation (naive) and 48 h of LPS stimulation (inflammatory) stained with DAPI (blue) and anti-SGPP-1 (green) (*n* = 7). (c, d) Representative pictures of confocal laser microscopy of untreated mature Flt3-differentiated CD103^+^ dendritic cells after 24 h of starvation (naive) and 24 h of LPS stimulation (inflammatory) stained with DAPI (blue) and anti-SGPP-1 (green) (*n* = 2). (e) Representative Western blot stained with anti-SGPP-1 antibody and anti-*β*-actin antibody in crude cytosolic (Cyt/Mic/Ly; Cyt = cytosol, Mic = microsomes, and Ly = lysosomes) and crude nuclear (cNun) fraction of cell lysates of naive GM-CSF-differentiated dendritic cells or 48 h upon LPS stimulation (*n* = 3). (f) Quantification of Western blot bands of anti-SGPP-1 staining after stimulation with LPS.

Since SGPP-1 protein levels in whole cell lysates tended to increase in inflammatory DCs while SGPL-1 concentration decreased, we next examined levels in more detail. Using confocal fluorescence microscopy we found that in resting, starving DCs, a large proportion of anti-SGPP-1 staining was localized within the nuclei and only a small quantity was present in the extranuclear compartment (Figure 2(a)). In contrast, in inflammatory DCs, the majority of the anti-SGPP-1 staining was no longer present within the nuclei but was found in the cytoplasmatic compartment (Figure 2(b)). In a supporting experiment, we produced Flt3 ligand-differentiated CD103^+^ cDCs and stimulated them in a similar way. Also here, the inflammatory response was accompanied with the translocation of anti-SGPP-1 staining from the nucleus to the cytoplasm (Figures 2(c) and 2(d)). To further confirm these results, we isolated cellular fractions to separate crude nuclear fraction (majority nuclear protein) from cytosolic, lysosomal, and ER (referred to as cytosolic fraction). Western blot indicated SGPP-1 bands in both compartments. Notably, protein concentration was higher in crude nuclear extract than in the extranuclear compartment in naïve DCs. However, under inflammatory conditions, SGPP-1 protein levels were increased in the cytosolic fraction and decreased in the nuclear fraction. Thus, in comparison to naïve DCs, SGPP-1 protein levels diminished upon LPS stimulation in the nucleic compartment (Figures 2(e) and 2(f)).

**Figure 3:**
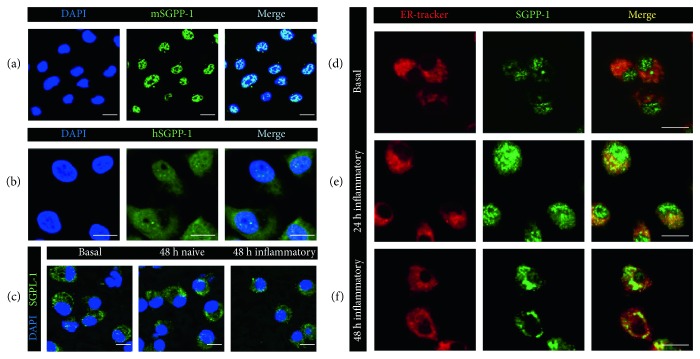
(a–f) Representative pictures of confocal laser microscopy of (a) untreated mature GM-CSF-differentiated murine bone marrow-derived dendritic cells stained with murine anti-SGPP-1 antibody (green) and DAPI (blue) (*n* = 5), (b) untreated mature human monocyte-derived dendritic cells stained with human anti-SGPP-1 antibody (green) and DAPI (blue) (*n* = 3), (c) untreated mature murine GM-CSF-differentiated dendritic cells (basal), after 48 h of starvation (naive) and 48 h of LPS stimulation (inflammatory) stained with DAPI (blue) and anti-SGPP-1 antibody (green) as indicated (*n* = 3), and (d–f) mature GM-CSF-differentiated murine dendritic cells stained with ER tracker (pseudocolor red) and anti-SGPP-1 antibody (green) (d) under basal conditions, (e) after 24 h of LPS stimulation, and (f) after 48 h of LPS stimulation (*n* = 3).

Notably, we found that most of the endogenous anti-SGPP-1 staining was localized in the nuclei in mature untouched DCs seven days after differentiation (Figure 3(a)). Antibody specificity was confirmed by a preincubation of the corresponding blocking peptide prior to the staining and showed absent staining in the nuclei (Supplementary Figure 1). In additional support, we produced human monocyte-derived DCs in which nuclear location of SGPP-1 was also confirmed using a human anti-SGPP-1 antibody (Figure 3(b)). Anti-SGPL-1 staining was present solely in the cytoplasmic compartment. Compared to SGPP-1, a similar change in SGPL-1 protein localization was not detected upon stimulation (Figure 3(c)).

To make further clarification on the translocation of SGPP-1 in inflammatory DCs, we costained cells with an ER tracker and found that the majority of the translocated protein was present in the ER compartment upon stimulation (Figures 3(d), 3(e), and 3(f)).

### 3.3. Extracellular Sphingosine Is Increased during Inflammatory Responses of Dendritic Cells

**Figure 4:**
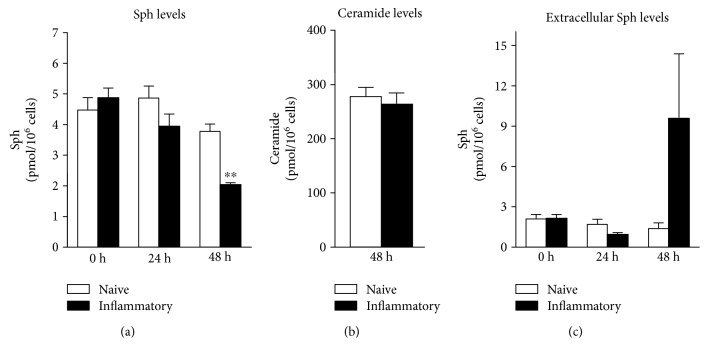
Quantification of sphingolipids and ceramides with LS-MS/MS normalized to 10^6^ cells (a) intracellular sphingosine levels, (b) intracellular ceramide levels (C14-C24), and (c) extracellular sphingosine levels (*n* = 4). Data represent mean ± SD; ^∗∗^*p* ≤ 0.01.

Since we detected an upregulation of SGPP-1 protein in the cytosolic fraction, we asked if fading S1P levels that we have observed under inflammatory conditions [2] are due to its increased dephosphorylation by SGPP-1 resulting in the formation of sphingosine. Thus, we next determined sphingosine levels within DCs. However, we found that with time sphingosine did not accumulate in the cells but diminished (Figure 4(a)). To investigate if sphingosine was converted to ceramide species instead, we determined intracellular C14-C24 ceramide levels. Although ceramide levels in sum were much higher than sphingosine levels, no differences in the single species between naïve and inflammatory DCs were observed (presented as whole ceramide levels in Figure 4(b)). To elucidate if sphingosine was released from the cells, we furthermore quantified extracellular lipids and found an increase of sphingosine levels, possibly indicating that a proportion of sphingosine generated by SGPP-1 was released into the medium of inflammatory DCs (Figure 4(c)).

### 3.4. Spns2 Is Not Responsible for S1P Export from Dendritic Cells

**Figure 5:**
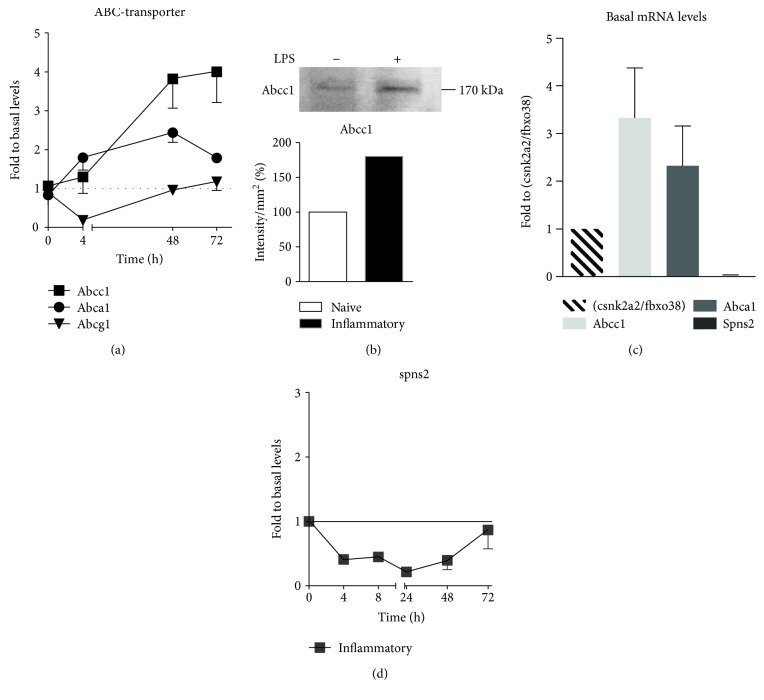
(a) Quantitative real-time PCR for abcc1, abca1, and abcg1 in LPS-stimulated dendritic cells over time normalized to the sum of csnk2a2 and fbxo38 mRNA levels. Levels at time point 0 were set to 1 for each mRNA (*n* = 4). (b) Western blot stained with anti-Abcc1 antibody of whole-cell lysates of naïve dendritic cells or 48 h upon LPS stimulation. Quantification of Western blot bands (*n* = 1). (c) Quantitative real-time PCR for abcc1, abca1, and spns2 in untreated dendritic cells (*n* = 4). Displayed are absolute levels normalized to the sum of csnk2a2 and fbxo38 mRNA levels. (d) Quantitative real-time PCR for spns2 in LPS-stimulated dendritic cells over time normalized to the sum of csnk2a2 and fbxo38 mRNA levels. Levels at time point 0 were set to 1 (*n* = 4). Data represent mean ± SD.

In this context, the question arose whether S1P is also actively transported outside the cell by transporters causing its intracellular decrease. Therefore, we concentrated on the regulation of known S1P transporters. An ATP-binding cassette (ABC) transporter array was performed and revealed the presence of several ABC transporters in dendritic cells (Supplementary Figure 2). The three prominent hits, namely *abca1*, *abcc1*, and *abcg1* were analyzed further. While *abcg1* was downregulated upon long-term inflammatory conditions, *abca1* and *abcc1* mRNA were upregulated (Figure 5(a)). Abcc1 was also upregulated on protein level upon 48 h of inflammatory stimulation possibly contributing to S1P fate (Figure 5(b)). A major S1P transporter, spinster homologue 2 (spns2) [19], was marginally expressed compared to absolute levels of selected ABC transporters (Figure 5(c)). No upregulation was observed in inflammatory DCs (Figure 5(d)). Extracellular S1P level determination in an adapted and upscaled cellular setting compared to conventional experiments was performed to overcome detection limits by LC-MS/MS. Analysis indicated that no accumulation of S1P was present in the medium upon stimulation (Supplementary Figure 3).

## 4. Discussion

As we and others have published before, the central immunological function of dendritic cells is dependent on both the S1P receptor-controlled modulation of key cytokines and the basic cellular survival or apoptosis mediated by sphingolipid metabolizing enzymes [2, 3, 20, 21]. In detail, different from model cells of sphingolipid research ranging from yeast to HEK293, in primary lymphocytes and as shown here in dendritic cells, there is no straightforward relationship of interdependent S1P synthesizing versus S1P-degrading enzymes. Therefore, with the current investigation, we aimed to elucidate this phenomenon in more detail. Indeed, our data confirmed a quite complex sequence of S1P and sphingosine rheostat with corresponding sphingolipid enzyme and transporter expression. As one example of this enigmatic intracellular S1P situation, we observed that intracellular S1P levels in inflammatory-activated DCs are fading despite the downregulation of SGPL-1 protein and enzyme activity. Although this downregulation could be a compensatory mechanism for progressive S1P loss, we conclude that its contribution to decreasing S1P levels in this context is rather small. Instead, we could assign an important role in S1P fate towards SGPP-1. Although mRNA levels of *sgpp-1* declined through stimulation, we here show that protein levels rather increased in the cytosolic compartment. This opposite regulation of mRNA levels could indicate another compensatory mechanism for the increasing amount of protein or fading S1P levels but might also hint towards different half-lives of mRNA and protein thus not reflecting the actual scope of the translational process.

SGPP-1 was described to colocalize with the ER and Golgi in MCF7 cells, HEK293 cells, and NIH 3T3 fibroblasts upon transient expression of SGPP-1 constructs [12, 22, 23]. In this study, we demonstrate for the first time the endogenous localization of SGPP-1. In differentiated DCs, endogenous SGPP-1 is localized mostly in the nucleus. Upon stress situation like starvation and even more under inflammatory conditions, SGPP-1 is translocated from the nucleus to the cytosolic compartment, where colocalization with the ER was finally observed. Thus, we conclude that the systematic shift of SGPP-1 from the nucleus to the cytoplasm contributes to the metabolism of S1P into sphingosine and thus loss of cytoplasmic S1P. It was presented before that SGPP-1-mediated S1P metabolism leads to the production of ceramide rather than an accumulation of sphingosine in mSGPP-1-transfected HEK293 cells [22, 23]. In our inflammatory dendritic cells, sphingosine was not accumulating either; however, the mean of ceramide levels did not change between naïve and inflammatory conditions. Since ceramide levels in dendritic cells were much higher than sphingosine levels, a direct metabolism might however not be visible. Notably, a portion of sphingosine was released into the extracellular space, and thus, we hypothesize that increased metabolism of S1P by cytosolic SGPP-1 is at least partially leading to a release of sphingosine.

A release of S1P into the extracellular space might demonstrate another route of disposing S1P. It was reported that S1P is exported, for example, from erythrocytes and platelets in an ATP-dependent manner [24, 25]. Several publications showed or postulated the transport of S1P by specific ABC transporters, for example, by Abcc1 in antigen-activated mast cells [26–28]. An upregulation of Abcc1 was also described in SGPL-1-deficient fibroblasts [29]. We similarly observed an upregulation of Abcc1 in DCs upon TLR4 activation which might contribute to S1P fate in inflammatory DCs. However, in our studies, extracellular S1P did not increase. Besides ABC transporters, spns2 is a prominent S1P transporter in endothelial cells [30]. However, herein, we additionally show that spns2 does not play a discriminate role in DCs since it was only marginally expressed and not upregulated upon inflammatory stimulation. Similarly, spns2 absence has been shown for blood cells before [31].

## 5. Conclusion

This study resulted in three major new findings. First, we demonstrate and propose that the major proportion of endogenous SGPP-1 is located in the nuclear compartment in murine- and human-differentiated dendritic cells. Second, upon inflammatory stimuli, translocation of SGPP-1 into the ER occurs and potentially contributes to S1P fate and sphingolipid rheostat in dendritic cell immune response. Third, spns2 is only marginally expressed in dendritic cells and is rather not relevant for S1P transportation in inflammatory dendritic cell.

## Supplementary Materials

Supplementary 1 Figure 1: Representative pictures of confocal microscopy of GM-CSF-differentiated dendritic cells after (a) anti-SGPP1 staining (*n* = 7) and (b) anti-SGPP1 staining upon 30 min preincubation with SGPP-1 blocking peptide (1:2) (*n* = 2) and (c) secondary antibody staining control (*n* = 7).

Supplementary 2 Figure 2: ABC transporter Array (*n* = 1).

Supplementary 3 Figure 3: Quantification of extracellular S1P levels by LC-MS/MS (*n* = 3).
