# Supplementary figures and images for: Nuclear Translocation of SGPP-1 and Decrease of SGPL-1 Activity Contribute to Sphingolipid Rheostat Regulation of Inflammatory Dendritic Cells
Source: Mediators Inflamm. 2017 Dec 11;2017:5187368. doi: 10.1155/2017/5187368 (PMC5742514; doi:10.1155/2017/5187368)

Supplementary 1

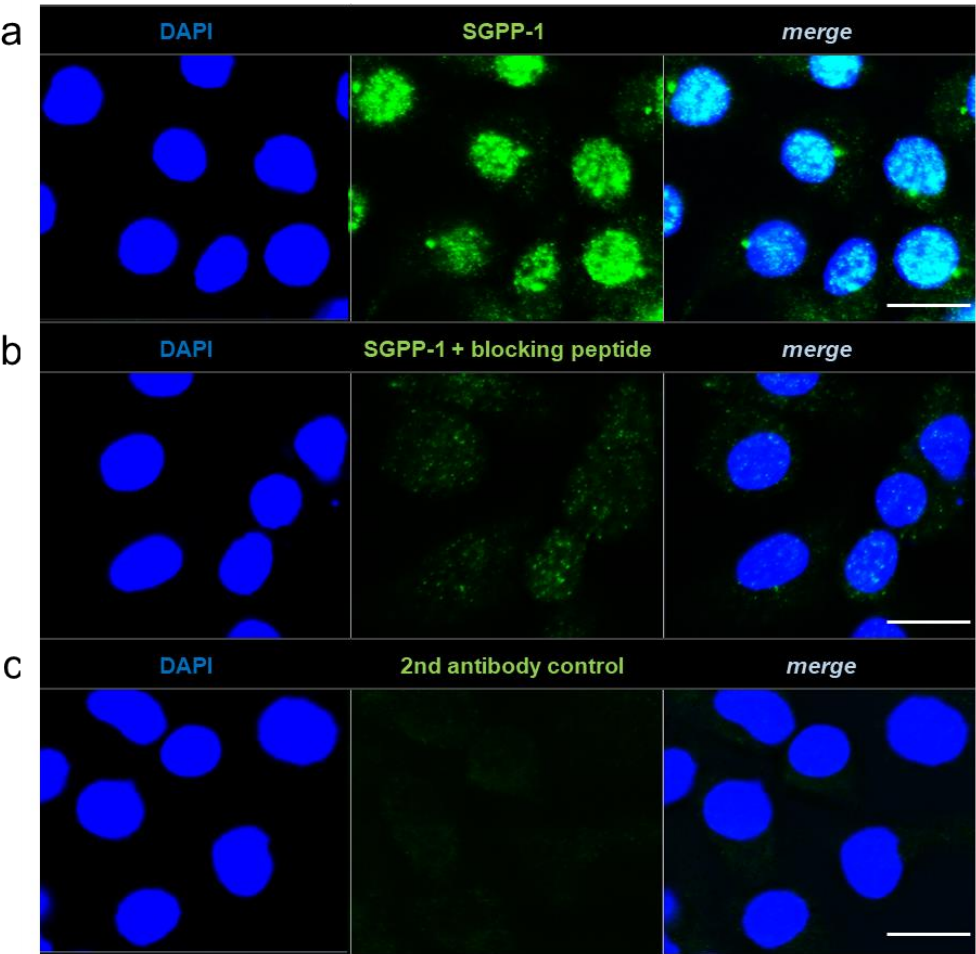

Supplement: Supplementary 1 — Figure 1: Representative pictures of confocal microscopy of GM-CSF-differentiated dendritic cells after (a) anti-SGPP1 staining (n = 7) and (b) anti-SGPP1 staining upon 30 min preincubation with SGPP-1 blocking peptide (1:2) (n = 2) and (c) secondary antibody staining control (n = 7). [file 5187368.f1.pdf]

## Supplementary 2

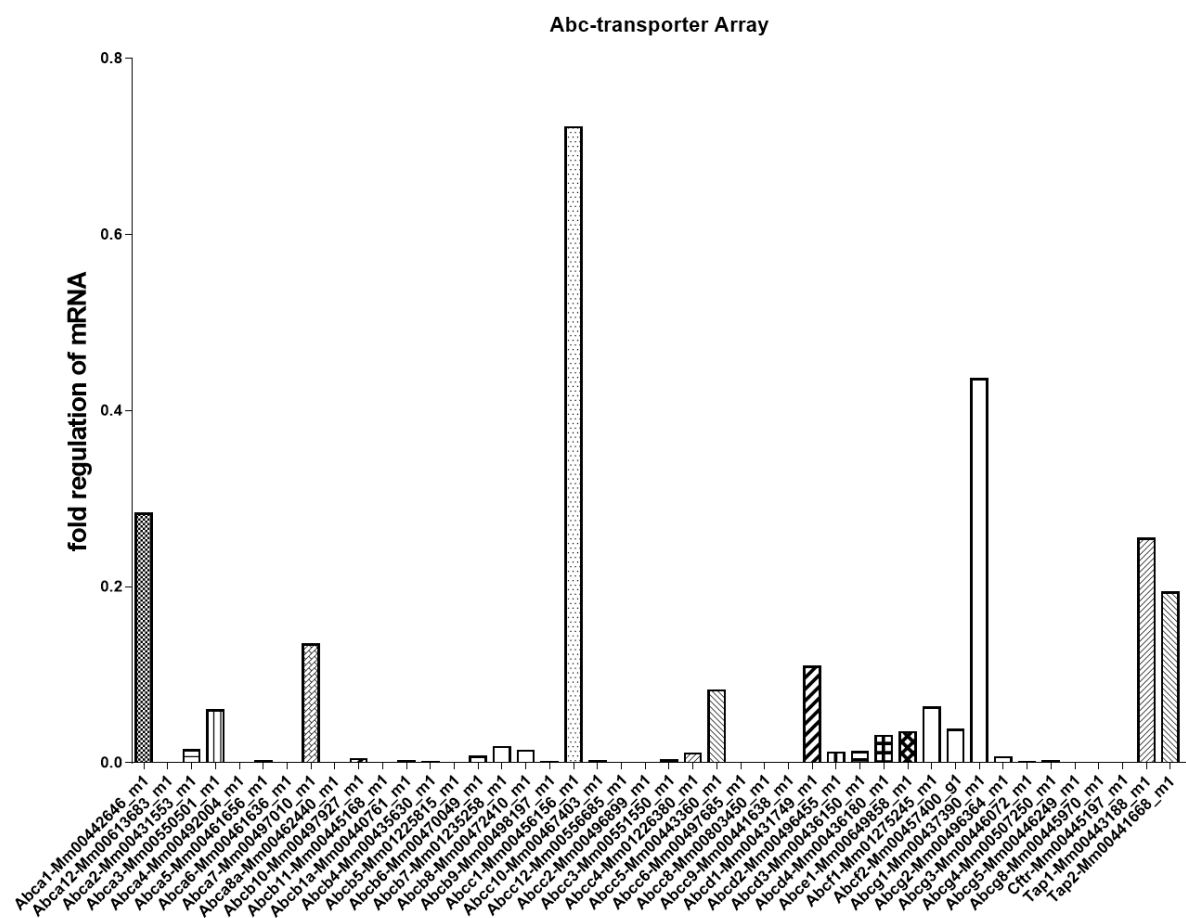

Supplement: Supplementary 2 — Figure 2: ABC transporter Array (n = 1). [file 5187368.f2.pdf]

Supplementary 3

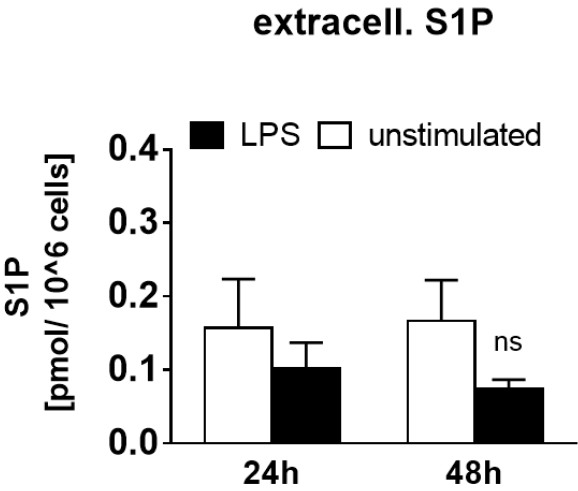

Supplement: Supplementary 3 — Figure 3: Quantification of extracellular S1P levels by LC-MS/MS (n = 3). [file 5187368.f3.pdf]
